# Multi-repeat sequences identification using genome mining techniques for developing highly sensitive molecular diagnostic assay for the detection of *Chlamydia trachomatis*

**DOI:** 10.12688/openresafrica.14316.1

**Published:** 2024-01-08

**Authors:** Clement Shiluli, Shwetha Kamath, Bernard N. Kanoi, Racheal Kimani, Michael Maina, Harrison Waweru, Moses Kamita, Ibrahim Ndirangu, Hussein M. Abkallo, Bernard Oduor, Nicole Pamme, Joshua Dupaty, Catherine M. Klapperich, Srinivasa Raju Lolabattu, Jesse Gitaka

**Affiliations:** 1 Centre for Research in Infectious Diseases, College of Graduate Studies and Research, Mount Kenya University, Thika, Kiambu County, Kenya; 2 Division of Research and Development, Jigsaw Bio Solutions Private Limited, Bangalore, India; 3 Animal and Human Health Program, International Livestock Research Institute, Nairobi, Nairobi County, Kenya; 4 Department of Materials and Environmental Chemistry, Stockholm University, Stockholm, Stockholm County, Sweden; 5 Department of Biomedical Engineering, Boston University, Boston, Massachusetts, USA

**Keywords:** Identical Multi-Repeat Sequences, Chlamydia trachomatis, Isothermal assays

## Abstract

*Chlamydia trachomatis* ( *C. trachomatis*) is a common sexually transmitted infection (STI). In 2019, the World Health Organization reported about 131 million infections. The majority of infected patients are asymptomatic with cases remaining undetected. It is likely that missed *C. trachomatis* infections contribute to preventable adverse health outcomes in women and children. Consequently, there is an urgent need of developing efficient diagnostic methods. In this study, genome-mining approaches to identify identical multi-repeat sequences (IMRS) distributed throughout the *C. trachomatis* genome were used to design a primer pair that would target regions in the genome. Genomic DNA was 10-fold serially diluted (100pg/mL to 1×10 ^-3^pg/mL) and used as DNA template for PCR reactions. The gold standard PCR using 16S rRNA primers was also run as a comparative test, and products were resolved on agarose gel. The novel assay, *C. trachomatis* IMRS-PCR, had an analytical sensitivity of 4.31 pg/µL, representing better sensitivity compared with 16S rRNA PCR (9.5 fg/µL). Our experimental data demonstrate the successful development of lateral flow and isothermal assays for detecting *C. trachomatis* DNA with potential use in field settings. There is a potential to implement this concept in miniaturized, isothermal, microfluidic platforms, and laboratory-on-a-chip diagnostic devices for reliable point-of-care testing.

## Introduction

*Chlamydia trachomatis* ( *C. trachomatis*) is among the most common sexually transmitted infections (STIs) that lead to adverse birth and neonatal outcomes such as pre-term labor and low birth weight infants ^1^. During delivery, the risk of mother-to-child transmission of *C. trachomatis* increases ^2^. Most patients with *C. trachomatis* infections are asymptomatic, and these cases remain undetected and untreated, further complicating the management of infected cases in most countries ^3^. It is, therefore, most likely that undiagnosed maternal *C. trachomatis* infections may lead to unavoidable adverse health outcomes in women and children worldwide, for example, salpingitis, endometritis, tubo-ovarian abscesses, pelvic peritonitis, and perihepatitis ^4^. In 2019 the World Health Organization (WHO) reported about 131 million infections of *C. trachomatis* globally ^5^.

Traditional methods of *C. trachomatis* testing include cervical cytological examination using Papanicolaou (Pap) smears ^6^. However, these techniques have many challenges, such as technical difficulties during cultures, labor intensiveness, increased turnaround time, and high cost. *C. trachomatis* infections are diagnosed by indirect and direct methods ^7^.

Direct methods detect the presence of *C. trachomatis* in localized infections ^7^. These methods include culture, antigen tests (Enzyme Immuno Assays (EIA), Direct Fluorescent Antibody (DFA) tests, immune chromatographic tests, Rapid Detection Tests (RDTs), and Nucleic Acid Amplification Tests (NAATs)) ^7^. Indirect methods are specific to *C. trachomatis* antibodies and are used for diagnostic evaluation of chronic or invasive infection (Pelvic Inflammatory Disease and lymphogranuloma venereum) and post-infectious complications, like sexually acquired reactive arthritis (SARA) ^7^.

Direct detection methods, such as NAATs, have several advantages. They use polymerase chain reaction (PCR) and use fluorescent-labeled probes to identify amplification products in real-time, and these reduce the turn-around time ^8^. Studies have also shown that *C. trachomatis* PCR using DNA extracted from conjunctival swabs can achieve a detection limit of up to 100 plasmid copies ^9^.

Other NAATs, such as ligase chain reaction and transcription mediated amplification, are also specific and sensitive in the screening of *C. trachomatis* in patient samples ^10^. Results of these tests can be generated in a few hours when coupled with automated nucleic acid extraction techniques.

However, NAATs such as the Abbot Real-Time CT/NG requires stringent sample transport and storage conditions ^11^. For example, to achieve accurate and reliable results, samples from asymptomatic women must be stored between 2°C and 30°C and processed within 14 days after collection ^11^. Symptomatic women's specimens must be thawed, frozen, and stored at the same temperature range ^11^. These conditions may be challenging, mainly in resource-limited countries where refrigeration facilities are unavailable, especially during specimen collection and transport ^12^.

When compared to *C. trachomatis* culture, the DFA has a sensitivity of between 95–100% ^13^. However, DFA involves labor-intensive microscopic examination of individual stained specimens, which can be time-consuming when dealing with many samples ^13^. Also, highly skilled and experienced personnel perform routine microscopic examinations ^13^. By contrast, EIAs are suitable for testing of many samples and have sensitivities of 90% as compared to culture, however, they are less accurate than DFA and test results may be false positive ^13^.

Thriving *C. trachomatis* culture is dependent on isolated live organisms, and cases identified in clinical samples ranges between 60–80%. In addition, culture sensitivity can be affected by improper specimen sample collection, and transport, storage, toxic material in patient samples, and colonization of cell cultures by opportunistic microorganisms ^14^. *C. trachomatis* culture has prolonged turn-around time; it is labor intensive, and different laboratories have various standardization protocols ^14^.

Indirect methods used for the identification of *C. trachomatis* are inaccurate in identifying acute infections of the lower reproductive and digestive tract; this is because antibody responses become detected after several weeks to months after an initial infection ^14^.

Enzyme linked immuno-sorbent assays that detect bacterial lipopolysaccharide may cross-react with other gram negative bacteria, which may give false-positive results. The *C. trachomatis* antibody response may be absent or delayed in some patients, which makes many serological tests inaccurate ^15^. In addition, most of the *C. trachomatis* infections are asymptomatic and are diagnosed late, resulting in uninterrupted transmission ^16^. To manage asymptomatic *C. trachomatis* infections, there is an urgent need to develop efficient methods of diagnosis with appropriate specificity and sensitivity that can be incorporated as part of routine screening programs, can be used to identify new infections and prevent the transmission of *C. trachomatis* cases ^17^. In this study, we have developed a highly sensitive molecular method that uses *de novo* genome mining approaches to detect identical multi-repeat sequences (IMRS) in *C. trachomatis* bacterial genome to be used as both isothermal amplification and PCR assays. The assay has a potential for field deployability due to inherent high sensitivity.

## Methods

### Identical Multi-Repeat Sequence (IMRS) genome-mining

The primers used in this study were developed using Identical Multi-Repeat Sequence (IMRS) genome mining algorithm. The IMRS algorithm was designed by using the Java Collection Framework by plugging in Google Guava version 23.0-jre (open-source common libraries for Java; https://github.com/google/guava, last accessed September 1, 2018). In the *C. trachomatis* IMRS application, the algorithm performs an analysis of the genome to select similar repeating oligonucleotide sequences of various lengths. The algorithm fragments the *C. trachomatis* genome into cross-matching windows of size L and enumerates all fragmented L-mer sequences into a list along with their corresponding positional coordinates on the genome. The repeated L-mers are determined with their exact positions categorized and classified on the basis of the repeat count. The hit counts are then screened by calculating coordinates for a pair of repeat sequences that are next to each other on the *Chlamydia trachomatis* genome and within an amplifiable region for primer generation. The NIH’s Basic Local Alignment Search Tool (BLAST; NIH, Bethesda, MD; https://blast.ncbi.nlm.nih.gov/Blast.cgi, last accessed July 11, 2019) and the National Center for Biotechnology Information’s Primer-BLAST (NCBI, Bethesda, MD; https://www.ncbi.nlm.nih.gov/tools/primer-blast, last accessed July 11, 2019) evaluated the specificity of lead pairs and the best pair was then identified. To identify *C. trachomatis* IMRS primers, *C. trachomatis* genome was used as an input for IMRS algorithm; and the primer pair having maximum number of repeats was selected for assay design. BLAST analyses were then performed to ensure that the selected primer pair is specific only for *Chlamydia trachomatis* ^18^. The *C. trachomatis* genome (NCBI, NC_000117.1) was used as a standard to mine repetitive sequences of less than 30 bases that can serve as reverse or forward primers. The Basic Local Alignment Search Tool available from the NIH website was used to determine the primer sets to ascertain *C. trachomatis* specificity. The resulting primers were predicted to amplify various fragment sizes of DNA derived from different regions of the *C. trachomatis* genome.

Distribution of primer targets on the *C. trachomatis* genome was determined by the Circos plot version 0.69–9 (Circos; RRID:SCR_011798).

### *C. trachomatis* genomic DNA preparation

*C. trachomatis* DNA was purchased from the American Type Culture Collection (ATCC) (ATCC ^®^ VR-885D ^™^, LOT Number. 70013611) (Manassas, Virginia, USA) at an initial concentration of ≥1 × 10 ^5^ genome copies/μL. The original DNA stock concentration was diluted to 100 pg/μL (8.92 × 10 ^4^ copies/μL) and eventually diluted 100 fold and 10 fold in Tris EDTA buffer (Thermo Fisher Scientific, Waltham, Massachusetts, USA) for the respective PCR assays.

### 16S rRNA PCR

The 16S rRNA PCR assays were performed in a SimpliAmp Thermal Cycler (Applied Biosystems, Thermo Fisher Scientific, Waltham, Massachusetts, USA) in a 25 μL reaction volume composed of dNTPs (Thermo Fisher Scientific, Waltham, Massachusetts, USA) (0.2mM), reverse (AATCAATGCCCGGGATTGGT) and forward (TCCGGAGCGAGTTACGAAGA) primers (0.01 mM each) (Macrogen, Seoul, South Korea), Taq Hot-Start DNA polymerase (Thermo Fisher Scientific, Waltham, Massachusetts, USA) (1.25 U), and 1 μL *C. trachomatis* DNA. The thermocycling parameters were as indicated: 95°C for 3 min; 40 cycles of: 95°C for 30 s, 56°C for 30 s, 72°C for 30 s; and 72°C for 5 min and a final hold step of 4°C ^19^.

### *C. trachomatis* IMRS PCR

The *C. trachomatis* IMRS assays were carried out in a SimpliAmp Thermal Cycler (Applied Biosystems, Thermo Fisher Scientific, Waltham, Massachusetts, USA) in a 25 μL reaction volume consisting of dNTPs (Thermo Fisher Scientific, Massachusetts, USA) (0.2 mM), reverse (TGTAGGAGGAGCCTCTTAGAGAA) and forward (TGCTGCTGCTGATTACGAGCCGA) primers (0.01 mM) (Jigsaw Biosolutions, Bengaluru, India), Taq Hot-Start DNA polymerase (Thermo Fisher Scientific, Massachusetts, USA) (1.25 U) and 1 μL *C. trachomatis* DNA. The thermocycling parameters for *C. trachomatis*-IMRS PCR assay was as indicated: 95°C for 3 min; 40 cycles of: 95°C for 30 s, 50°C for 30 s, 72°C for 30 s; and 72°C for 5 min and a final hold of 4°C. All PCR amplicons were resolved in 1% agarose gel and visualized on a UV Gel illuminator machine (Fison Instruments, Glasgow, United Kingdom) under ethidium bromide staining ^19^.

### Isothermal IMRS amplification

The Isothermal IMRS amplification was done in a reaction volume of 25 μL composed of the following *Bst* 2.0 polymerase (640 U/mL) (New England Biolabs, Massachusetts, USA), with 1X amplification buffer, 1.6 μM reverse primer (TGTAGGAGGAGCCTCTTAGAGAA), and 3.2 μM forward primer (TGCTGCTGCTGATTACGAGCCGA) (Jigsaw Biosolutions, Bengaluru, India) with 10 mM dNTPs (Thermo Fisher Scientific, Massachusetts, USA), 0.4 M Betaine (Sigma-Aldrich, Missouri, USA), molecular-grade water and Ficoll (0.4 g/mL) (Sigma-Aldrich, Missouri, USA). Amplification was performed at 56°C for 40 min. PCR amplicons were resolved in 1% agarose gel and visualized on a UV Gel illuminator machine (Fison Instruments, Glasgow, United Kingdom) under ethidium bromide staining ^19^. 

### Lower limit of detection

To determine the lower limit of detection (LLOD), DNA template was diluted 100-fold from 100 pg/μL (8.92 × 10 ^4^ copies/μL) to 10 ^-6 ^pg/μL (<1 copies/μL) and 10-fold from 100 pg/μL (8.92 × 10 ^4^ copies/μL) to 10 ^-2^ pg/μL (< 1 copies/μL) for the *C. trachomatis* IMRS PCR and gold standard 16S rRNA PCR, respectively. Five replicates of each dilution were then used for the PCR assays. PCR amplicons were resolved in 1% agarose gel and visualized on a UV Gel illuminator machine (Fison Instruments, Glasgow, United Kingdom) under ethidium bromide staining. To calculate the LLOD of the 16S rRNA and IMRS *C. trachomatis* PCR, probit analysis was done by determining the ratio of reactions that were successful to the total number of reactions performed. Similarly, to assess the LLOD for the *C. trachomatis* Isothermal IMRS PCR assay, genomic DNA was diluted 10-fold from a starting concentration of 1.64 × 10 ^6^ copies/μL. Thereafter, the LLOD was calculated as mentioned earlier ^19^.

### *C. trachomatis*-Lateral Flow Assay

To produce a visual read-out signal of amplified products, the *C. trachomatis*-Lateral Flow Assay ( *C. trachomatis*-LFA) was performed as follows in a final master mix volume of 25 μL, 2.5 μL annealing buffer, dNTPs and NaCl (1.75 μL), MgSO _4 _(1.2 μL), NG 5’ biotinylated forward primer (Biotin 5'-“TGCTGCTGCTGATTACGAGCCGA”-3'), *C. trachomatis* Reverse primer, *C. trachomatis* 3’ FAM labelled probe (CCACCAATACTCTC/-FAM-3’), *C. trachomatis* Digotexin labelled probe, Ficoll 400 (6.25 μL), internal control sequence (2.5 μL), ISO Amp III enzyme mix 2.0 μL, *C. trachomatis* DNA 5μL and molecular grade water 1.45 μL. The LFA strip (Milenia Biotec GmbH, Giessen, Germany) were incubated at 65°C for 1 hour, thereafter, 5 μL of reaction liquid was added to the LFA strip and 2 drops of buffer added ^19^.

### Real-time PCR assay

Real-Time PCR assay was performed on the QuantStudio™ 5 Real-Time PCR System (Applied Biosystems, Thermo Fisher Scientific, Waltham, Massachusetts, USA) as a comparative method ^20^ for determining the sensitivity of the *C. trachomatis* IMRS and 16S rRNA PCR primers for detecting *C. trachomatis* DNA. The genomic DNA was serially diluted 10-fold starting concentration of 10 ^4^ genome copies/μL. The PCR was done in triplicate in a final master mix volume of 10 μL and was composed of the following: 1 μL reverse and forward IMRS primer mix, 5 μL SYBR Green qPCR Master Mix (Thermo fisher, Massachusetts, USA), 2.5 μL *C. trachomatis* DNA and 1.5 μL molecular grade water. The thermocycling conditions were 50°C for 2 min; 95°C for 10 min; 40 cycles of 95°C for 15 s and 60°C for 30 s.

### Confirmation of *C. trachomatis*-IMRS amplicons using gene cloning

Specificity *C. trachomatis*-IMRS primers was also confirmed using gene cloning. Amplification of *Chlamydia trachomatis* gDNA was done using Assembly IMRS-F (ttccggatggctcgagtttttcagcaagattgcctgccT **GCTGATTACGAGCCGA**) and Assembly IMRS-R (agaatattgtaggagatcttctagaaagatt **GTAGGAGGAGCCTCTTAGAGAA**) primer set. The bold and underlined sequences represent the IMRS primers specific for the *C. trachomatis* genome whereas the non-priming overlap lowercase sequence at the 5 ´-end of the primers sequence corresponds to the homologous sequences in the vector used for cloning. The PCR products were resolved on 2% agarose to ascertain the size of the fragment and thereafter the PureLink™ PCR purification kit (ThermoFisher) was used for purification. The NEBuilder® HiFi DNA Assembly kit (NEB) was used to ligate the PCR products into pJET1.2 blunt vector (ThermoFisher) following the manufacturer’s protocol. The NEBuilder HiFi DNA Assembly product obtained was further transformed into NEB 5-alpha Competent *E. coli* (NEB #C2987, NEB) following the manufacturer’s recommendations. Colonies that were transformed were selected randomly, DNA extraction and Sanger-sequencing done using the universal pJET1.2 forward sequencing primer (cgactcactatagggagagcggc) and pJET1.2 reverse sequencing primer (aagaacatcgattttccatggcag). The nucleotides obtained were trimmed and assessed using SnapGene sequence analysis software (Version 6.1 GSL Biotech; available at snapgene.com, RRID:SCR_015052), alignment performed to determine similarity and or clonal differences and BLAST used assess for similarity with the *C. trachomatis* genomic sequences.

### Clinical samples

Vaginal swab samples collected from a cohort of women aged between 19 – 49 years participating in an STI study in Nairobi County at the Kenyatta National Hospital (KNH) in 2022 were used to validate the *Chlamydia trachomatis* IMRS PCR method. Enrolled participants provided written informed consent. This study was approved by the KNH Ethics Review Committee on the 13 ^th^ of April 2022 (P928/12/2021). The *C. trachomatis*-16S rRNA PCR method was used to screen for *C. trachomatis* cases. Thereafter, positive clinical samples were used to validate the *C. trachomatis*-IMRS primers. Participants were notified of results directly and confidentially by study staff, and were treated for *C. trachomatis* infection. The Mount Kenya University Ethical Review Committee (MKU/ERC/1649) approved the study and use of clinical samples on the 23 ^rd^ of October 2020.

### Data analysis

The mean, and SD values calculation, and graphs were done using Microsoft Excel (RRID:SCR_016137) 2016. Probit regression analyses and calculation of the LLOD of *C. trachomatis*-IMRS and *C. trachomatis*-16S rRNA PCR assays (the concentration at which *Chlamydia trachomatis* DNA is determined with 95% confidence), was done in Excel 2016. *Trichomonas vaginalis* and *Treponema pallidum* DNA were used to confirm the specificity of the *C. trachomatis*-IMRS primers for other related infections. Analyses were done using paired *t*-test for two-tailed distribution. *P* < 0.05 was considered statistically significant.

## Results

### Development and IMRS primer distribution targets on *Chlamydia trachomatis* genome

**Figure 1.**
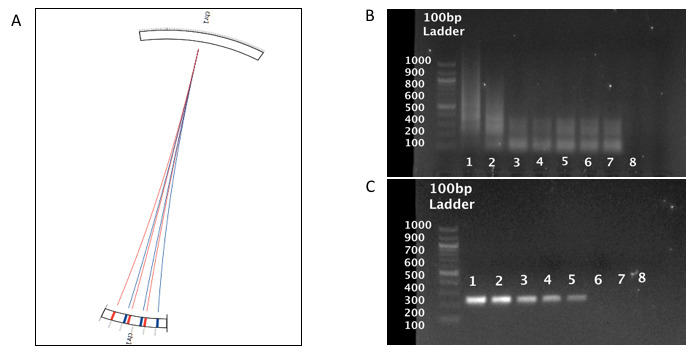
*Chlamydia trachomatis*-IMRS primer targets and gel images from the *C. trachomatis* IMRS and conventional 16S rRNA PCR assay. ( **A**) Circos plot for the distribution of identical multi repeat sequence (IMRS) primers in the *C. trachomatis* genome. *C. trachomatis* IMRS primer A (blue lines) IMRS primer B (red lines) both have 6 repeats. Image of 10-fold serially diluted (1 – 100, 2 – 10, 3 – 1, 4 – 0.1, 5 – 0.01, 6 – 0.01, 7 – 0.001 and 8 - NTC) (pg/μl) genomic *C. trachomatis* DNA amplicons resolved on 1% gel using IMRS primers ( **B**) and gold standard 16S rRNA PCR ( **C**).

Using the IMRS-based genome mining algorithm ^10^, six repeat sequences ( Table 1) ^19, 21^ were identified on the *Chlamydia trachomatis* genome, which could serve as reverse and forward primers for the PCR assay. The primers (F 5’- TGCTGCTGCTGATTACGAGCCGA -3’ and R 5’- TGTAGGAGGAGCCTCTTAGAGAA - 3’), as were depicted using a Circos plot version 0.69–9 (Circos; RRID:SCR_011798) ( Figure 1), are present at a number of loci allowing them to serve interchangeably as reverse or forward primers.

**Table 1.** IMRS Primer target regions on the *Chlamydia trachomatis* bacterial genome. IMRS, identical multi-repeat sequences.

No.	Amplification regions	Expected sequence fragment
1	531375 - 531511	137bp
2	531375 - 531661	287bp
3	531375 - 531814	440bp
4	531525 - 531661	137bp
5	531525 - 531814	290bp
6	531675 - 531814	140bp

### Specificity of *C. trachomatis* identical multi-repeat primers

**Figure 2.**
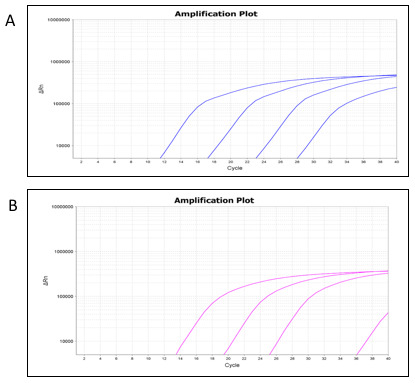
Real-time PCR amplification plots from the *Chlamydia trachomatis*-identical multi repeat sequence (IMRS) ( **A**) and the 16S rRNA ( **B**) assays using serially diluted *C. trachomatis* genomic DNA.

To confirm specificity of the IMRS primers for regions on the *C. trachomatis* genome, serially diluted DNA was used as template for PCR amplification. The IMRS primers amplified *C. trachomatis* DNA at a starting template concentration of 0.1 fg/μL ( Figure 1B), and the gold standard 16S rRNA primers ( Figure 1C) detected *C. trachomatis* DNA to a concentration of 10 fg/μL. This shows that the IMRS-PCR assay has a 100-fold higher sensitivity compared to the conventional 16S rRNA PCR assay. Diluted *C. trachomatis* DNA was used as a template for real-time PCR amplification using *C. trachomatis*-IMRS primers ( Table 2A), and conventional *C. trachomatis*-16S rRNA primers ( Table 2B). Amplification curves were plotted using the mean Ct values at each respective dilution ( Figure 2A, *C. trachomatis*-IMRS primers and Figure 2B, *C. trachomatis*-16S rRNA primers).

**Table 2.** Genomic DNA dilution to determine the sensitivity of the *C. trachomatis*-IMRS primers using Real time PCR. IMRS, identical multi-repeat sequences.

A, Serially diluted *Chlamydia trachomatis* genomic DNA served as amplification templates for the *C. trachomatis*-IMRS primers		
Concn of DNA (genome copies/ *µ*l)	Ct value	STD.DEV
1×10 ^4^	12.298	0.541
1×10 ^3^	17.674	1.244
1×10 ^2^	23.639	0.349
1×10 ^1^	28.265	0.275
B, Serially diluted *Chlamydia trachomatis* genomic DNA served as amplification templates for the 16S rRNA primers		
Concn of DNA (genome copies/ *µ*l)	Ct value	STD.DEV
1×10 ^4^	14.64	0.902
1×10 ^3^	20.584	1.599
1×10 ^2^	26.607	1.056
1×10 ^1^	36.527	0.249

### Determination of the Lower Limit of Detection (LLOD)

**Figure 3.**
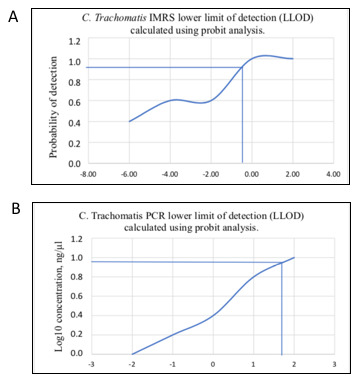
Probit regression analysis to estimate the lower limit of detection for the *Chlamydia trachomatis*-identical multi repeat sequence (IMRS) primers and the 16S rRNA PCR. Probit analysis estimation for *C. trachomatis*-IMRS ( **A**). As indicated, the IMRS primers for *C. trachomatis* had an LLOD = 9.5 fg/μl. **B**: Probit analysis estimation for 16S rRNA PCR. As indicated, gold standard primers for *C. trachomatis* had an LLOD = 4.31 pg/μl.

To calculate the lower limit of detection (LLOD) of the IMRS PCR and the conventional 16S rRNA PCR, probit statistic was calculated using concentrations of 100 and 10 fold serially diluted *C. trachomatis* DNA and thereafter the dilutions served as starting template for the *C. trachomatis*-IMRS and 16S RNA PCR respectively ( Table 3A and Table 3B). The probit plots for the *C. trachomatis*-IMRS PCR and the gold standard 16S rRNA PCR assay are shown in Figure 3A and Figure 3B. The concentration at which *C. trachomatis* DNA can be estimated with 95% confidence was used to calculate the LLOD. Probit calculation for the *C. trachomatis*-IMRS PCR, had a coefficient of χ = -3.6494 and a *P*-value of 0.7043 ( Table 4). As shown, the *C. trachomatis* IMRS primers had a detection limit of 9.5 fg/μL ( Figure 3A). The probit calculation for the 16S rRNA PCR is shown in Table 4, and had a coefficient of χ = -7.2101 and a *P*-value of 0.9978. The 16S rRNA PCR assay for *C. trachomatis* had an LLOD of 4.31 pg/μL ( Figure 3B). In summary, the *C. trachomatis*-IMRS PCR assay was more sensitive than the gold standard 16S rRNA PCR assay.

**Table 3.** *Chlamydia trachomatis* genomic DNA was serially diluted 100 folds (A) and 10-folds (B) and used as template for the *C. trachomatis*-IMRS and 16S rRNA PCR to estimate the lower limit of detection.

A, 100-fold dilution	
Serial dilutions (pg/μl)	Replicates (5)
100	5/5
1	5/5
0.01	4/5
0.0001	5/5
0.000001	0/5
0.00000001	0/5
B, 10-fold dilution	
Serial dilutions (pg/μl)	Replicates (5)
100	5/5
10	4/5
1	3/5
0.1	1/5
0.01	1/5
0.001	0/5

**Table 4.** Shows the statistics obtained from the Probit analysis. IMRS, identical multi-repeat sequences.

Assay	χ Coefficient	P-Value
16S rRNA	-7.2101	0.9978
IMRS	-3.6494	0.7043

### Genomic *Chlamydia trachomatis* DNA amplification using the Isothermal assay

**Figure 4.**
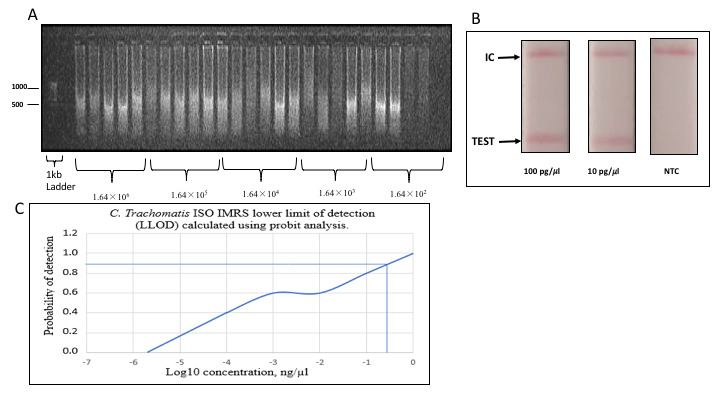
*Chlamydia trachomatis*-Iso-identical multi repeat sequence (IMRS), lateral flow assay and estimation of the lower limit of detection of the isothermal assay. ( **A**) Gel image of *C. trachomatis*-Iso IMRS assay products visualized on 1% gel. Five replicates of each dilution served as DNA template for the *C. trachomatis*-Iso IMRS. DNA concentration is in genome copies per μl. ( **B**) Visual read-out detection of serially diluted *C. trachomatis* DNA using the lateral flow assay. Amplicons were incubated at 65°C for 1 hour and transferred onto strips as indicated. IC – Internal Control, NTC – Non Template Control. ( **C**) Probit analysis estimation for *C. trachomatis* Iso-IMRS. As indicated, the IMRS primers for *C. trachomatis* had an LL0D = 0.3162 ng/μl.

Serially diluted genomic DNA was used to perform the Isothermal- *C. trachomatis*-IMRS amplification and the resulting reaction products visualized on a 1% gel ( Figure 4A). The Isothermal- *C. trachomatis*-IMRS assay successfully amplified *C. trachomatis* DNA down to 1.64×10 ^2^ genome copies/μL. The LLOD for the *C. trachomatis*-Iso-IMRS assay was estimated at 0.3162 ng/μL ( Figure 4C).

### *Chlamydia trachomatis* Lateral Flow Assay

A visual readout signal was observed when serially diluted DNA was transferred on lateral flow assay (LFA) strips ( Figure 4B). The LFA readout of the amplification products was successful indicating the potential and applicability of the Isothermal-IMRS assay in the field.

### Plasmid *C. trachomatis*-DNA concentration in ng/μl of transformed *E. coli* cells

**Figure 5.**
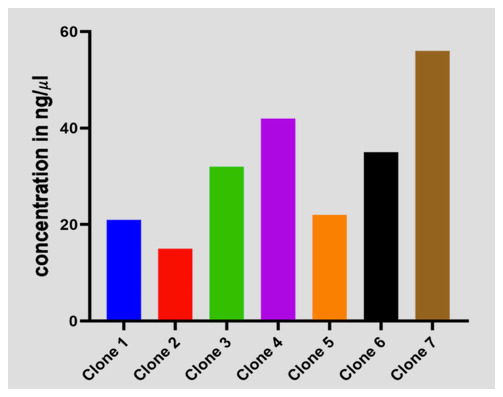
Transformed *E.coli* cells expressing *Chlamydia trachomatis* sequences amplified using *C. trachomatis*-identical multi repeat sequence (IMRS) primers.

To validate the exact regions that were amplified by the *C. trachomatis*-IMRS primers, we cloned and transformed the amplicon into blunt vectors into electrocompetent *E. coli* cells, thereafter, the clones were plated on agar plates. Transformed cells were selected from eight colonies ( Figure 5) and DNA extracted and sanger sequencing performed. Multiple sequence alignment confirmed *C. trachomatis* sequences. These shows that the *C. trachomatis*-IMRS primers are specific for targets within the *C. trachomatis* genome.

### Validation of *C. trachomatis*-IMRS primers

**Figure 6.**
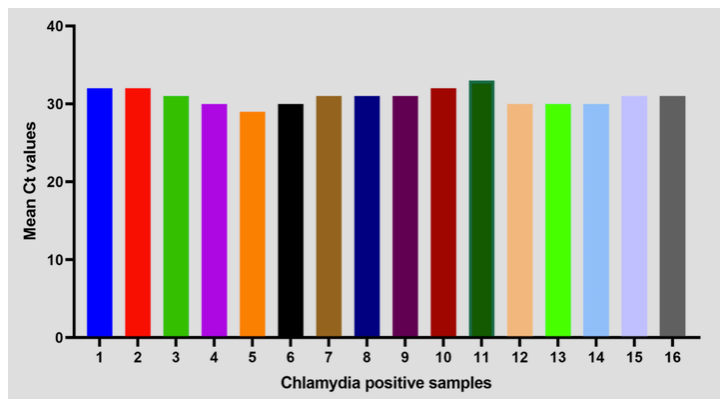
Mean Ct values of PCR confirmed 16 clinical DNA samples from RT-PCR assay that were used to validate the *Chlamydia trachomatis*-identical multi repeat sequence (IMRS) PCR primers.

*Chlamydia trachomatis* positive DNA samples from a cross-sectional study at the Kenyatta National Hospital were used to validate the *C. trachomatis*-IMRS primers for identifying *C. trachomatis* DNA using RT-PCR assay as indicated in Figure 6. The demographic information for recruited participants has been described in Table 5. Results from RT-PCR assay using *C. trachomatis*-IMRS primers were concordant with the results from conventional PCR.

**Table 5.** Demographic data for participants.

Variable		Chlamydia positive, n = 16,(%)	Chlamydia negative, n = 187, (%)	P-Value
*Age Category*				
	10 – 19	0, 0	2, 1	0.9467
	20 – 29	6, 38	63, 34	
	30 – 39	9, 56	105, 56	
	40 – 49	1, 6	17, 9	
*Marital status*				
	Married	13, 81	161, 86	0.5949
	Not Married	3, 19	26, 14	
*Level of Education*				
	Primary	1, 6	14, 7	0.5013
	Secondary	8, 50	66, 35	
	Tertiary	7, 44	107, 57	
*Employment status*				
	Employed	8, 50	55, 29	0.0875
	Not employed	8, 50	132, 71	
*Parity*				
	Primipara	1, 6	39, 21	0.3702
	Multipara	14, 88	138, 74	
	Grandmultipara	1, 6	10, 5	
*Gestational Age*				
	First trimester	0, 0	11, 6	0.0637
	Second trimester	7, 44	37, 20	
	Third trimester	9, 56	139, 74	
*HIV Status*				
	Positive	1, 6	1, 1	0.0263
	Negative	15, 94	186, 99	
*Miscarriage*				
	None	9, 56	111, 59	0.8421
	Once	5	52, 28	
	Twice	2	14, 7	
	Thrice	0, 0	7, 11	
	Quadruple	0, 0	3, 2	

### Specificity and sensitivity of the *C. trachomatis*-IMRS primers

**Figure 7.**
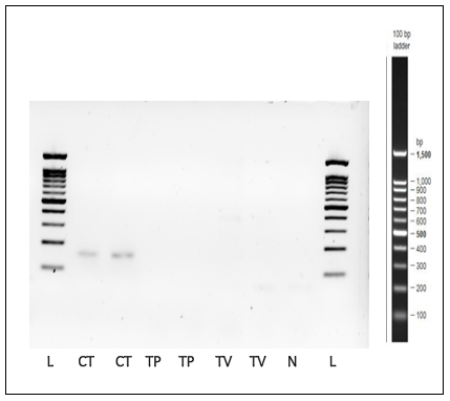
Gel image of PCR products obtained after amplification of *Treponema pallidum* (TP) and *Trichomonas vaginalis* (TV) genomic DNA using *Chlamydia trachomatis*-identical multi repeat sequence (IMRS) primers. *C. trachomatis* genomic DNA was used as a positive control. N = Negative control, L = Ladder.

As indicated in Figure 7, *C. trachomatis*-IMRS primers showed specificity to *C. trachomatis* DNA and non-specific to *Trichomonas vaginalis* and *Treponema pallidum* genomic DNA. Compared to the conventional 16S-rRNA PCR, the *C. trachomatis*-IMRS PCR accurately detected *C. trachomatis* genomic DNA at starting template concentration of 1 fg/μl ( Figure 1B).

## Discussion

Traditional techniques for identifying *C. trachomatis* infections are less sensitive, require collection of invasive patient samples, have complex algorithms and test result reporting, and are expensive ^17^. As a consequence, there is need of developing novel tests that are sensitive, specific and readily applicable in field set-ups. In the present study, we demonstrate the use of a deep genome mining strategy to identify identical multiple repeat sequences that could be used as robust primers for a novel nucleic acid-based test that is a highly sensitive test against *C. trachomatis*. Specifically, IMRS forward and reverse primers are able to initiate amplicon generation at various loci on the *C. trachomatis* genome. Therefore, the overall analytical sensitivity is improved by producing many amplicons ^22, 23^. Our findings confirm that, compared to the gold standard 16S rRNA PCR, using IMRS primers to amplify of specific sequences on the *C. trachomatis* genome is sensitive, yielding large number of amplicons of different sizes hence a lower detection limit of 9.5 pg/mL (8.4 genome copies/mL). Our results are comparable to the Chlamydial Roche Amplicor Real-Time Quantitative PCR with a lower limit of detection of 200 genome copies/ml ^24^. However, this assay targets up to 10 copies of chlamydial plasmid.

We also confirmed that isothermal amplification of DNA using IMRS PCR primers is reliable and sensitive for detecting *C. trachomatis*. The *C. trachomatis*-Iso-IMRS assay detected DNA up to 8.9 genome copies per mL. The Isothermal-IMRS assay had increased sensitivity compared to a Loop-mediated isothermal assay utilizing the ompA and orf1 genes, which reported a detection limit of 50 copies per mL ^25^.

Our study had success in developing and testing a Lateral Flow Assay technology to detect *C. trachomatis* to a concentration of 10 pg/mL (8.8 genome copies/ml) ( Figure 6). Our finding was different from a study that developed a visual read-out of *C. trachomatis*-LAMP method based on a gold nanoparticle lateral flow biosensor that reported a limit of detection of 50 copies/ml after an incubation of 45 minutes ^26^.

Compared to nucleic acid methods that are used for the identification of *C. trachomatis* that are mostly suboptimal, the *C. trachomatis*-IMRS PCR primers were specific and sensitive when used for the identification of *C. trachomatis* DNA.

## Conclusions

Put together; here we show that the IMRS algorithm can serve as a platform technology for designing primers that are sensitive and specific for *C. trachomatis*. This platform has potential application in other bacterial and non-bacterial pathogens and could significantly improve future disease diagnostics procedures.

## Data Availability

Zenodo: Chlamydia trachomatis RAW DATA. https://zenodo.org/doi/10.5281/zenodo.10200809 ^19^. GenBank: Chlamydia trachomatis D/UW-3/CX, complete genome. Accession number https://identifiers.org/ncbi/insdc:AE001273.1 ^21^. Data are available under the terms of the Creative Commons Attribution 4.0 International license (CC-BY 4.0).
