# Supplementary material for: Multi-repeat sequences identification using genome mining techniques for developing highly sensitive molecular diagnostic assay for the detection of Chlamydia trachomatis
Source: Open Res Afr. 2024 May 9;7:2. Originally published 2024 Jan 8. [Version 2] doi: 10.12688/openresafrica.14316.2 (PMC11109563; doi:10.12688/openresafrica.14316.2)
Supplement: Peer review history

# Reviewer report: Reviewer response for version 2

**DOI:** 10.21956/openresafrica.16048.r31268

**Published:** 2024-05-21

**Authors:** Shehu Awandu

**Affiliations:** 1 Jaramogi Oginga Odinga University of Science and Technology, Bondo, Kenya

The authors have adequately responded to my comments

Is the work clearly and accurately presented and does it cite the current literature?

Yes

If applicable, is the statistical analysis and its interpretation appropriate?

Yes

Are all the source data underlying the results available to ensure full reproducibility?

Yes

Is the study design appropriate and is the work technically sound?

Yes

Are the conclusions drawn adequately supported by the results?

Yes

Are sufficient details of methods and analysis provided to allow replication by others?

Yes

Reviewer Expertise:

NA

I confirm that I have read this submission and believe that I have an appropriate level of expertise to confirm that it is of an acceptable scientific standard.

# Reviewer report: Reviewer response for version 1

**DOI:** 10.21956/openresafrica.15473.r30839

**Published:** 2024-03-23

**Authors:** Tom Were

**Affiliations:** 1 Masinde Muliro University of Science and Technology, Kakamega, Kakamega County, Kenya

Summary:

 The study developed a highly sensitive molecular method based on de novo genome mining to detect multi-repeat sequences (IMRS) in *C. trachomatis* genome for isothermal amplification and PCR assays. The IMRS identified 6 repeat sequences that generated forward and reverse sequence primers for PCR yielding amplicons of 137-440bp. The primers also had high ct values at amplification of 16s rRNA in serially diluted (1x10 ^4^-1x10 ^1^) genomic DNA copies. The study also identified that the primers gave a lower limit of detection of 0.001 pg/ml at 100-fold, and 0.01 at 10-fold genomic DNA dilution. In addition, cloning validation experiments indicated that the primers had a LLOD of 9.5 fg/ml vs. gold standard of 4.5 pg/ml. Specificity and sensitivity evaluations show that the primers were specific for *Chlamydia vaginalis* bacteria. However, it is important that the authors add specificity and sensitivity chi-square. calculations. The study identified and validated through IMRS-technology and PCRs primers with potential for detection of *C. trachomatis* infections in patients.

 Additional comments:

- Paragraph #1: line 10-14 – also add the fact that C. trachomatis is a major cause of infertility through blocked fallopian tubes.
- Paragraph #4-6, should be moved to after paragraph #10 with line 8-18 of current paragraph #10 moving to the new paragraph #10.
- Specify the number of clinical samples used in the validation of the assay
- Methods – page 4, line 24: indicate the target/band sizes
- Methods – page 4, line 41: interchange the order of reverse and forward primers as well as in line 53
- Methods – indicate the source of ethidium bromide in the whole chapter
- Table 5 is not described in the text
- Discuss the limitations of the study
- In the conclusion you state that the primers were sensitive and specific, yet sensitivity and specificity including AUC were not analyzed.

Is the work clearly and accurately presented and does it cite the current literature?

Yes

If applicable, is the statistical analysis and its interpretation appropriate?

Yes

Are all the source data underlying the results available to ensure full reproducibility?

Yes

Is the study design appropriate and is the work technically sound?

Yes

Are the conclusions drawn adequately supported by the results?

Partly

Are sufficient details of methods and analysis provided to allow replication by others?

Yes

Reviewer Expertise:

Infectious diseases; genetics; laboratory medicine; and immunology

I confirm that I have read this submission and believe that I have an appropriate level of expertise to confirm that it is of an acceptable scientific standard.

# Reviewer report: Reviewer response for version 1

**DOI:** 10.21956/openresafrica.15473.r30594

**Published:** 2024-03-01

**Authors:** Shehu Awandu

**Affiliations:** 1 Jaramogi Oginga Odinga University of Science and Technology, Bondo, Kenya

Describe the advantages of the new techniques besides sensitivity. Turn around time? Indicate, qPCR  assay efficiency etc. Specific treatment offered to the infected patients. Figure 1 B & C are incomplete. PCR bands are fuzzy, were assays optimised? Show sensitivity and specificity in % when compared to the gold standard.

Is the work clearly and accurately presented and does it cite the current literature?

Yes

If applicable, is the statistical analysis and its interpretation appropriate?

Yes

Are all the source data underlying the results available to ensure full reproducibility?

Yes

Is the study design appropriate and is the work technically sound?

Yes

Are the conclusions drawn adequately supported by the results?

Yes

Are sufficient details of methods and analysis provided to allow replication by others?

Yes

Reviewer Expertise:

NA

I confirm that I have read this submission and believe that I have an appropriate level of expertise to confirm that it is of an acceptable scientific standard, however I have significant reservations, as outlined above.
